# Nécrose pariétale utérine partielle après capitonnage hémostatique au cours d'une hémorragie de la délivrance

**DOI:** 10.11604/pamj.2013.15.32.2327

**Published:** 2013-05-25

**Authors:** Fatima Zohra Fdili Alaoui, Sofia Jayi, Hakima Bouguern, Moulayabdilah Melhouf, Hinde El Fatemi, Afaf Amarti

**Affiliations:** 1 Department Of Gynecolgy And Obstetrics Ii, Chu Hassan Ii, Fez, Morocco; 2 Department Of Anatomic Pathology, Chu Hassan Ii, Fez, Morocco

**Keywords:** Hémorragie de la délivrance, Atonie utérine, capitonnage utérin, nécrose utérine, Postpartum hemorrhage, uterine atony, uterine padding, uterine necrosis

## Abstract

L'hémorragie de la délivrance reste la première cause de mortalité dans la majorité des pays y compris le Maroc. Sa prise en charge doit être multidisciplinaire, rapide, avec mise en ‘uvre de moyens médicaux et parfois chirurgicaux pour contrôler l'hémorragie. Les techniques de compression ou de cloisonnement utérins ont été peu évaluées mais diffusées rapidement dans le monde entier vu la facilité de leur réalisation; Néanmoins des complications secondaires ont été rapportées dans la littérature dont la nécrose utérine. Nous rapportons le cas d'une patiente de 33ans césarisée à 38 semaines d'aménorrhée pour utérus cicatriciel associé à une grossesse gémellaire avec inertie utérine. Un capitonnage hémostatique a été nécessaire pour contrôler l'hémorragie, l’évolution a été marquée par la survenue d'une nécrose partielle utérine confirmée histologiquement. Nous insistons à travers cette observation et sous la lumière de la revue de la littérature sur la nécessité d'un suivi post opératoire des patientes qui bénéficient de capitonnage hémostatique pour mieux documenter l'efficacité et les complications de cet acte chirurgical qui reste encore sous évalué.

## Introduction

Les techniques chirurgicales de compression utérine font parie de la stratégie thérapeutique adoptée face à l'hémorragie de la délivrance, leur diffusion rapide est due à leur facilité de réalisation. Cependant certaines complications secondaires dont la nécrose utérine ont été rapportées dans la littérature. Nous rapportons un nouveau cas d'une patiente présentant une nécrose utérine partielle post capitonnage.

## Patient et observation

**Figure 1:**
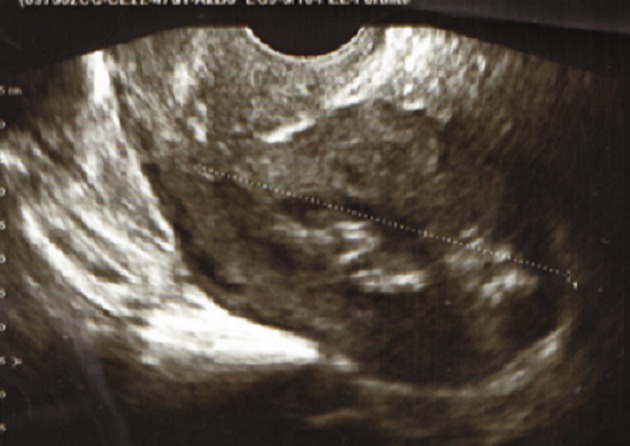
Échographie pelvienne objectivant une ligne d'interface interrompue au niveau du corps utérin par une image hétérogène faisant suspecter une rétention placentaire

**Figure 2:**
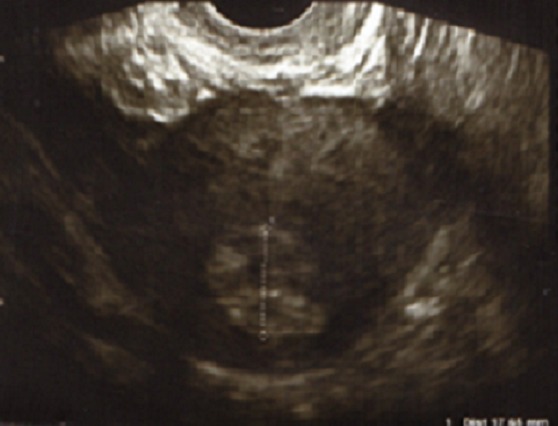
Échographie pelvienne objectivant une image hétérogène intracavitaire mesurant 18mm faisant suspecter une rétention placentaire

Il s'agit de Mme RK, âgée de 33 ans, troisième geste ayant comme antécédent une césarienne lors de sa première grossesse il y a 4 ans pour dépassement de terme, une fausse couche curetée à 2 mois. La grossesse actuelle a été suivie dans notre formation. Une césarienne a été programmée à 38 semaines d'aménorrhée pour utérus cicatriciel +grossesse gémellaire laquelle a permis l'extraction de deux nouveaux nés de sexe masculins pesant 2600g et 3000grames, mais a été compliquée par la survenue d'une inertie utérine. Le massage utérin complété par la perfusion d'ocytocine et l'admission de 5comprimés en intrarectal de misoprostol (vu la non disponibilité de sulprostone) n'ont pas corrigé l'atonie utérine d'où le recours à la triple ligature de Tsirulnikov, ligature des artères hypogastriques et capitonnage par deux points simples utérins verticaux et corporéaux(Vicryl1, aiguille ronde, demi-cercle, 40 mm, Stericat Gutstrings(P) Ltd), ce qui a permis l'arrêt de l'hémorragie; la patiente a été transfusée par 4 culots globulaires, 2 culots plaquettaires et 4 plasma frais congelé. Le taux d'hémoglobine est passé de 11, 2 à 9, 4 g/dl en post-opératoire. Les suites opératoires ont été sans particularité, et la patiente a été déclarée sortante à j+4 sous traitement martial, contraception microprogestative vu le désir d'allaitement et hystéroscopie diagnostique pour évaluation de la cavité utérine prévue à 6 mois. A 2 mois du post partum, la patiente consulte pour persistance de métrorragies noirâtres minimes, une échographie pelvienne (Figure 1, Figure 2) a objectivé une ligne d'interface interrompue au niveau du corps utérin par une image hétérogène mesurant 18mm faisant suspecter une rétention placentaire. La patiente a été mise sous méthylergométrine sans amélioration. L'hystéroscopie a montré des lambeaux effilochés de couleur blanchâtres avec au niveau du fond utérin une image jaunâtre sphacélée; le tableau évoquant une rétention trophoblastique. Plusieurs biopsies sous contrôle visuel ont été réalisées au niveau des zones suspectes avec tentative d'extraction à la pince. Macroscopiquement, ces fragments correspondaient à du myomètre nécrosé sans mise en évidence ni de caduque ni d’éléments trophoblastiques et le diagnostic final retenu était celui d'une nécrose utérine partielle.

## Discussion

L'hémorragie de la délivrance est la première cause de mortalité maternelle au Maroc [1], c'est une urgence obstétricale qui met rapidement en jeu le pronostic vital maternel. Son traitement classique consiste en un certain nombre de gestes (massage utérin, révision utérine, sondage vésical à demeure, examen de la filière génitale, perfusion d'ocytocine, voire de prostaglandines) et des mesures de réanimation (remplissage vasculaire, transfusion de concentrés globulaires) qui devront être réalisées systématiquement avant d'envisager une escalade thérapeutique. En cas d’échec de ces mesures, et/ou si l’état de la patiente est instable, un traitement chirurgical s'impose [2]. De nombreux progrès ont intéressé le traitement conservateur (ligatures vasculaires, plicatures et capitonnages utérins), le traitement chirurgical radical (hystérectomie d'hémostase) reste parfois l'ultime geste pour sauver la malade.

Les techniques de plicature utérine de B-Lynch et de compression ou capitonnage utérin (Cho) apparaissent comme une alternative intéressante surtout en cas d'atonie utérine, elles permettent de réaliser une hémostase en comprimant la paroi antérieure contre la paroi postérieure par des points transfixiants simples ou en cadre; Ces techniques sont certes prometteuses mais méritent d'avantage de recul et de cas rapportés [3].

Malgré le fait que ces techniques de compressions ou de cloisonnements utérins ont été peu évaluées, leur facilité de réalisation a permis leur diffusion rapide dans le monde entier. En conséquent, certaines complications sont apparues: pyrométrie, érosion de la bretelle à travers le mur utérin, ischémie utérine, nécrose utérine, synéchie. Néanmoins la fréquence de ces complications reste difficile à déterminer vu l'absence de larges séries rapportées dans la littérature concernant ces procédures; elle pourrait être de 5à7% [4, 5].

**Figure 3:**
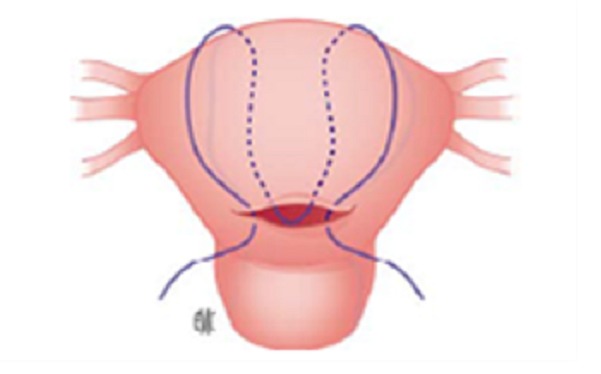
Compression myométriale en bretelle selon B-Lynch et al

**Figure 4:**
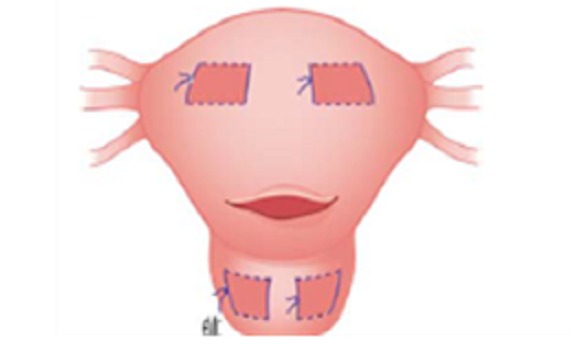
Capitonnage utérin en cadre selon Cho et al

La technique de B-Lynch consiste à réaliser une suture en bretelle autour du corps utérin (Figure 3), alors que la technique décrite par Cho consiste en des sutures multipoints en cadre (Figure 4) [6, 7]. La technique de B-Lynch a déjà fait l'objet de plusieurs publications rapportant des complications tel que la nécrose utérine partielle contrairement à la technique de Cho beaucoup moins documentée mais qui commence à être associée à ces complications dont la nécrose utérine [8].

La nature des fils utilisés (durée de résorption) et le degré de tension initial des points sont deux éléments pouvant expliquer les variations en termes d'ischémie. La technique en elle-même pourrait avoir une influence sur la survenue de la nécrose: une compression uniforme n'interrompant pas la vascularisation pariétale en totalité (notamment en évitant les sutures dans le sens horizontal et en les réalisant seulement dans les sens vertical) pourrait diminuer ce risque [9].Il semblerait que la mise en place correcte des points de compression (seule ou en association avec d'autres procédures hémostatiques) de telle sorte que la reperfusion du myomètre par le réseau anastomotique collatéral est préservée peut diminuer le risque de nécrose [10].

De nouvelles descriptions de sutures proposant des adaptations des techniques initiales sont publiées régulièrement révélant des avantages et des inconvénients à chacune [9]. Dans notre cas malgré la réalisation de points simples dans le sens vertical, nous avons eu comme complication la nécrose utérine partielle. La technique de plicature utérine et de compression ou capitonnage est un geste simple à réaliser. L’évaluation systématique de la cavité utérine après ce genre de traitement conservateur est nécessaire pour poser le diagnostic de nécrose utérine ou synéchie et pour éliminer une rétention placentaire qui constitue le principal diagnostic différentiel tant que l'innocuité quant au pronostic en termes de fertilité et fragilité utérine n'est pas établie.

## Conclusion

Le traitement conservateur par compression ou capitonnage utérin est un geste facile à réaliser s'intégrant dans la stratégie thérapeutique face à une hémorragie de la délivrance, mais dont l'innocuité par rapport à l'utérus n'est pas tout à fait établie. La nécrose utérine partielle constitue l'une des complications, ce qui nous pousse à faire une évaluation soigneuse de la cavité utérine à distance pour poser le diagnostic.
